# The interplay of microRNA and neuronal activity in health and disease

**DOI:** 10.3389/fncel.2013.00136

**Published:** 2013-08-27

**Authors:** Stephen M. Eacker, Ted M. Dawson, Valina L. Dawson

**Affiliations:** ^1^Neuroregeneration and Stem Cell Programs, Institute for Cell Engineering, Johns Hopkins University School of MedicineBaltimore, MD, USA; ^2^Department of Neurology, Johns Hopkins University School of MedicineBaltimore, MD, USA; ^3^Solomon H. Snyder Department of Neuroscience, Johns Hopkins University School of MedicineBaltimore, MD, USA; ^4^Department of Physiology, Johns Hopkins University School of MedicineBaltimore, MD, USA

**Keywords:** microRNA, synaptic plasticity, stroke, epilepsy, neuroprotective agents

## Abstract

MicroRNAs (miRNAs) are small 19–23 nucleotide regulatory RNAs that function by modulating mRNA translation and/or turnover in a sequence-specific fashion. In the nervous system, miRNAs regulate the production of numerous proteins involved in synaptic transmission. In turn, neuronal activity can regulate the production and turnover of miRNA through a variety of mechanisms. In this way, miRNAs and neuronal activity are in a reciprocal homeostatic relationship that balances neuronal function. The miRNA function is critical in pathological states related to overexcitation such as epilepsy and stroke, suggesting miRNA’s potential as a therapeutic target. We review the current literature relating the interplay of miRNA and neuronal activity and provide future directions for defining miRNA’s role in disease.

MicroRNAs are endogenously encoded small RNAs that are processed sequentially into mature 19–23 nucleotide (nt) regulatory molecules (Krol et al., 2010b). Once processed to their mature length, miRNAs are bound by the core component of the RNA-induced silencing complex (RISC), the PIWI-domain containing protein Argonaute (AGO). In mammals, there are four members of the Argonaute family (AGO1–4), all of which are capable of silencing mRNAs in a miRNA-dependent fashion. Of these AGO family members, only AGO2 is capable of directly catalyzing endonucleolytic cleavage of RNA targets, and only when there is complete complementarity between the miRNA and its target (Filipowicz et al., 2008). The AGO2-mediated cleavage of target RNAs is thought to be the primary effector of the exogenously supplied small interfering RNA (siRNA) or short hairpin RNA (shRNA). Most endogenous miRNA–mRNA interactions differ in two important ways (Bartel, 2009). First, most miRNAs share only partial complementarity with their mRNA targets, guiding RISC to its targets through interaction between the 5′-most 6–8 nt of the miRNA (the so-called seed sequence) and its mRNA target, usually in the 3′ untranslated region (3′ UTR). Second, though essential for miRNA-mediated silencing, AGO does not directly cleave mRNA targets. Instead, the AGO–miRNA complex recruits a host of additional factors that result in the silencing of mRNA targets.

The mechanism by which miRNAs silence their mRNA targets remains highly controversial (Filipowicz et al., 2008; Djuranovic et al., 2011). There are two primary camps in the mechanism of silencing debate. One camp believes that mRNA silencing by miRNAs occurs completely by mRNA deadenylation and decay. The other camp does not dispute that RISC can catalyze deadenylation and decay of targets, but believes there is an element of translational repression that precedes the destruction of target messages. Not surprisingly, there are excellent experiments that support both of these models that fuel this ongoing debate in the literature. This debate lies beyond the scope of this review, but remains important especially when considering how miRNAs sculpt neuronal function.

There are numerous ways in which miRNAs modulate the function of the nervous system. A variety of studies have shown how miRNA plays an important role in the development and in a wide array of disorders of the nervous system (Sun et al., 2013). The focus of this review is the interplay of miRNA and neuronal activity.

## miRNAs REGULATE STRUCTURAL AND SYNAPTIC PLASTICITY

**Figure 1:**
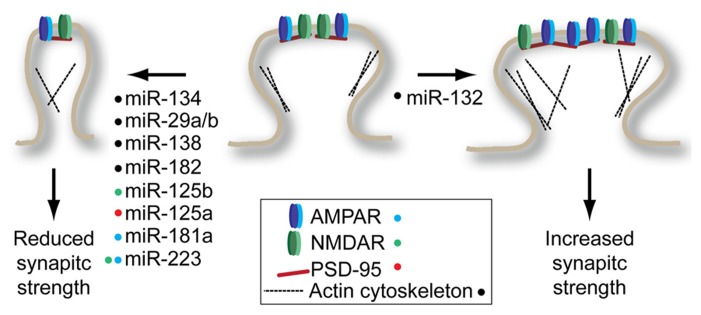
**miRNAs regulate structural and synaptic plasticity by targeting synaptic spine proteins.** Many miRNAs promote decrease in spine volume and/or glutamate receptor density (left) while only miR-132 has been shown to promote spine maturation (right). Colored dots next to miRNA indicate the target(s) of each miRNA shown to modulate plasticity. See text for details on individual miRNA targets.

Regulation of neuronal excitability is a major control point for synaptic plasticity, a fundamental component of learning and memory. Alterations in synaptic function are associated with both structural changes at the synapse and changes in synaptic strength (**Figure 1**). Structural changes at the synapse are commonly associated with alterations in the cytoskeletal function, leading either to the establishment or dissolution of synapses (Bosch and Hayashi, 2012; Rochefort and Konnerth, 2012). Altered synapse morphology can also reflect the strength and stability of a synapse owing to the associated size of the post-synaptic density, the site of synaptic transmission reception. In general, larger, mushroom-shaped spines are associated with larger post-synaptic densities, allowing for more stable and stronger synaptic transmission. This is in contrast to the more dynamic filopodia-type spines, which are associated with weak or absent synaptic transmission (Yoshihara et al., 2009).

One of the first synaptically enriched miRNAs identified, miR-134, was shown to be a negative regulator of synaptic spine volume (Schratt et al., 2006). This pioneering study showed that miR-134 acts by repressing Limk1 expression, a kinase that regulates spine morphology by regulating ADF/cofilin interactions with the actin cytoskeleton. The reduced spine volume associated with miR-134 overexpression should lead to reduced synaptic strength. Consistent with this hypothesis, mice overexpressing miR-134 show defects in the establishment of long-term potentiation (LTP) in the hippocampus (Gao et al., 2010). However, in this study the authors identified cAMP-response element binding protein (Creb) as a target of miR-134. It is conceivable that both studies found true, but different targets of miR-134: the low level of sequence complementarity required to guide a miRNA to its target means that every miRNA has potentially hundreds of targets. This highlights the somewhat arbitrary process of target identification for miRNAs when performing phenotypic analysis of miRNA overexpression or knockdown. Importantly, both studies found that inhibition of miR-134 increased levels of Limk1 and Creb, respectively, and reversed the synaptic and structural plasticity effects observed when miR-134 is overexpressed.

Structural plasticity-related mRNAs seem to be a prominent target for miRNAs. This observation is supported by a study by Chi et al. (2009) where neural miRNA targets were identified using an unbiased biochemical approach called high-throughput sequencing of RNAs isolated by crosslinking immunoprecipitation (HITS–CLIP). In this approach, the AGO protein was UV-crosslinked to its mRNA targets in a cell suspension derived from the cortex of young mice. The crosslinked mRNA–miRNA–AGO ternary complexes were immunoprecipitated and the RNAs from this purification were subject to high-throughput sequencing. Gene ontology analysis of target mRNAs revealed an enrichment of genes involved in cytoskeleton regulation, particularly overlapping with genes also identified as involved in neuronal differentiation.

The enrichment of miRNA targeting structural plasticity-related genes is found prominently throughout the literature. Actin related protein 2/3 complex, subunit 3 (Arpc3) was identified as a miR-29a/b target in a screen for miRNAs involved in drugs of abuse-related plasticity (Lippi et al., 2011). Overexpression of miR-29a/b increased filopodia-type spines and decreased mushroom-type spines. This spine phenotype is consistent with the phenotype observed when Arpc3 is knocked down. As predicted by the change in morphology of spines, there is a reduction in miniature excitatory post-synaptic current (mEPSC) frequency when miR-29a/b is overexpressed. The activity-induced miR-132 seems to have an opposite impact on structural plasticity. Knockdown of miR-132 activity in newborn neurons in the adult hippocampus or in neurons of the visual cortex leads to a reduction in stable mushroom spines and an increase in filopodia-type spines (Luikart et al., 2011; Mellios et al., 2011). These structural changes were accompanied by decreased frequency, but not amplitude of mEPSCs. Conversely, infusion of miR-132 mimics into the visual cortex following monocular deprivation increased mushroom-type spines and eliminated ocular-dominance associated plasticity (Tognini et al., 2011). The precise level of miR-132 seems to play an important role in plasticity – modest overexpression in the hippocampus improves performance in a Barnes maze test without altering spine density. However, when expressed at supraphysiological levels, miR-132 impairs performance in this memory task, which is accompanied by increased spine density (Hansen et al., 2013). It is worth noting that the target protein(s) that mediate this miR-132-dependent structural plasticity have yet to be identified.

Functional screening of synaptically enriched miRNA identified an interaction between miR-138 and the acyl protein thioesterase 1 (Apt1) mRNA (Siegel et al., 2009). Inhibition of miR-138 was the only synaptically enriched miRNA out of 11 tested to significantly increase the volume of dendritic spines of cultured hippocampal neurons. Surprisingly, this increased volume was accompanied by decreased mEPSC amplitude, which the authors ascribe to a decrease in GluR2 positive clusters found on the dendritic spines. Knockdown of the miR-138 target Apt1 recapitulates the effects on spine density observed in miR-138 overexpression, suggesting that miR-138 acts through Apt1 to regulate spine morphology.

The miRNA regulation of structural plasticity extends beyond the hippocampus and cortex. Auditory fear conditioning downregulates a number of miRNA in the amygdala (Griggs et al., 2013). Bioinformatic analysis suggested that of these downregulated miRNAs, miR-182 could bind to the 3′UTR of a number of key regulators of the actin cytoskeleton in synapses. Infusion of miR-182 mimics into the lateral amygdala led to downregulation of RAC1, cortactin, and to a lesser extent, cofilin. This same infusion protocol impaired long-term amygdala-dependent fear memory suggesting that the miR-182 plays a repressive role in memory formation. However, how precisely miR-182 impacts structural or synaptic plasticity remains to be determined.

In addition to regulating the structural aspects of dendritic spines, a number of miRNAs have been shown to directly regulate components of the post-synaptic density. The synaptically enriched miR-181a can target the GluR2 subunit of the 2-amino-3-(3-hydroxy-5-methyl-isoxazol-4-yl)propanoic acid receptor (AMPAR) through a conserved binding site in its 3′UTR (Saba et al., 2012). Interestingly, miR-181a is induced by dopamine D1/5 receptor agonist SKF-38393 *in vitro* and by amphetamine and cocaine in various anatomical structures *in vivo.* Surface levels of GluR2 are reduced in neurons overexpressing miR-181a; however, only mEPSC frequency and not amplitude is affected. The authors suggest that AMPAR-dependent spine development might be effected, explaining reduced mEPSC frequency. However, the possibility remains that miR-181a may have additional relevant targets in hippocampal neurons.

A number of mRNAs encoding post-synaptic density proteins appear to be shared targets of miRNAs and the fragile-X mental retardation protein (FMRP). The FMRP negatively regulates mRNA translation by directly interacting with target mRNAs. In one study, the authors identified miRNA enriched in FMRP-bound RNA immunoprecipitation experiments (Edbauer et al., 2010). Of these miRNAs enriched on FMRP-bound messages, miR-125b and miR-132 had significant effects on structural and synaptic plasticity when overexpressed. While miR-125b overexpression led to thinner spines and decreased amplitude of mEPSC, miR-132 overexpression led to the formation of short, thicker spines and increased mEPSC amplitude and frequency, roughly consistent with the miR-132 studies described above. Turning their focus to miR-125b, the authors identified the *N*-methyl-D-aspartate (NMDA) receptor 2A (NR2A) as a direct miR-125b target. Overexpression and knockdown of miR-125b had significant impact on the NMDAR currents, consistent with the observed up and downregulation of NR2A.

A second study found that the FMRP-bound PSD-95 transcript is under the regulation of miR-125a (Muddashetty et al., 2011). Interference with miR-125a induces increased PSD-95 and increased spine number. The FMRP-bound transcripts are translationally repressed and in many cases reactivated for translation by activity through the metabotropic glutamate receptors 1 and 5 (mGluR1/5; Darnell and Klann, 2013). Muddashetty et al. (2011) demonstrate that the PSD-95 mRNA is liberated from RISC following stimulation with the mGluR1/5 agonist dihydroxyphenylglycine (DHPG). Interestingly, both the study of Edbauer et al. (2010) and Muddashetty et al. (2011) found that FMRP was required for miRNA-mediated silencing of their targets. Although previous studies in *Drosophila* have shown physical interaction between RISC and FMRP (Ishizuka et al., 2002; Jin et al., 2004), current data suggest that mammalian RISC and FMRP do not physically interact. More likely, FMRP acts to create a permissive environment on the mRNA that allows RISC to bind and silence its targets. One way in which FMRP may accomplish this is by stalling translating ribosomes (Darnell et al., 2011), preventing ribosomes from dislodging RISC from coding sequence target sites. While most effective miRNA target sites are in the 3′UTR of genes, there is evidence of extensive RISC binding in coding regions (Chi et al., 2009; Helwak et al., 2013). Alternatively, FMRP binding in 3′UTRs could occlude the binding of other RNA-binding proteins that could otherwise dislodge RISC from its target.

## NEURONAL ACTIVITY ALTERS miRNA BIOGENESIS

**FIGURE 2:**
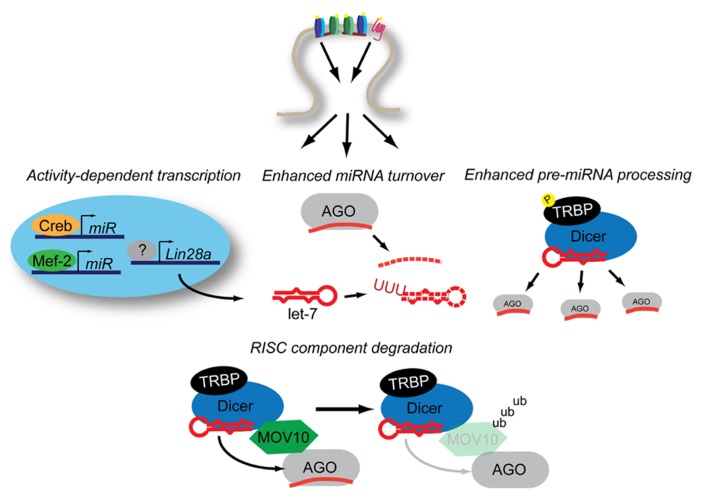
**Neuronal activity influences miRNA expression through multiple mechanisms.** Activity-dependent transcription induces miRNA production through Mef2 and Creb while Lin28a increases expression through an uncertain combination of transcription-dependent and -independent mechanisms. Increased levels of Lin28a lead to destabilization of pre-let-7 transcripts through poly-uridynylation. Activity also promotes accelerated miRNA turnover through an additional unknown mechanism(s). Activity-dependent phosphorylation of TRBP stabilizes Dicer, leading to increased mature miRNA production. NMDAR-activation promotes the ubiquitinylation of MOV10, a component of RISC, which may lead to decreased levels of mature-miRNA loaded RISC. See text for details.

The miRNA biogenesis is a multiple-step process that begins with the transcription of a primary miRNA transcript (pri-miRNA; Krol et al., 2010b). The pri-miRNA can be derived from a non-coding transcript containing one or many miRNAs, or can be processed from intronic sequences. A hairpin structure containing the mature miRNA sequence is recognized by the microprocessor complex, which cleaves the hairpin out of the context of the pri-miRNA transcript, yielding a miRNA precursor (pre-miRNA). The 60–80 nt pre-miRNA is processed by the RNase III protein Dicer to produce a double-stranded 19–23 bp miRNA. Then one strand is selectively loaded into AGO, yielding a mature miRNA engaged in RISC. Each of these steps are potential control points for the regulation of the cellular miRNA milieu. In this section, we will review how neuronal activity regulates the steps of miRNA biogenesis (**Figure 2**).

Activity has a strong influence on the transcriptional state of neurons. Creb is one of the primary transcriptional activators that respond to neuronal activity. In a genome-wide screen for Creb binding sites, Vo et al. (2005) identified two consensus binding sites near the miR-212/miR-132 locus. Stimulation of primary neurons with brain-derived neurotrophic factor (BDNF; Vo et al., 2005), KCl depolarization, or bicuculline (Wayman et al., 2008) all induce the production of pri-miR-132 transcript, making it the first recognized miRNA whose expression is regulated by neuronal activation. These observations have been supported by numerous *in vivo* investigations (Nudelman et al., 2010; Eacker et al., 2011; Mellios et al., 2011; Tognini et al., 2011; Wang et al., 2013) among others. Regulation through the cAMP response elements in the putative promoter region of the miR-212/miR-132 cluster were further confirmed by the activity-dependent acquisition of transcription-promoting chromatin marks in the visual cortex after visual stimulation (Tognini et al., 2011). Coupled with the potent effects of miR-132 on structural and synaptic plasticity (see above), it’s activity-dependent transcriptional control suggests that miR-132 is a potent regulator of experience-dependent plasticity *in vivo.*

A second, larger cluster of miRNAs, the miR-379~410 cluster, has also shown to be upregulated by neuronal activity (Fiore et al., 2009). This miRNA cluster is regulated both by BDNF and KCl depolarization via the myocyte enhancing factor 2 (Mef2) transcription factor. Among the many miRNAs transcribed from this locus is miR-134, a known regulator of structural plasticity through Limk1. In addition to directly regulating Limk1, this study suggests miR-134 may play a broader role in regulating protein synthesis by regulating the RNA binding protein Pumilio 2 (Pum2).

Increased expression of pri-miRNA transcript does not necessarily result in increased mature miRNA levels. One striking example of how neuronal activity can influence mature miRNA levels independently of transcription is through the induction the Lin28a RNA binding protein by BDNF (Huang et al., 2012). The Lin28 family members can selectively impair the processing of both pri- and pre-let-7 miRNA, though Lin28a primarily targets pre-let-7 through the post-transcriptional addition of poly-uridine to the 3′ end of the transcript (Thornton and Gregory, 2012). In response to bath application of BDNF in the presence of actinomycin D, hippocampal neurons show increased expression of Lin28a leading to a decrease in mature let-7 levels, resulting to increased translation of let-7 target RNAs. Under the same stimulating conditions, Huang et al. (2012) observed an increase in pre-miRNA processing of non-let-7 miRNA. This was the result of increased phosphorylation of the Dicer binding partner trans-activation response RNA-binding protein (TRBP). The extracellular signal-regulated kinase (ERK)-dependent phosphorylation of TRBP stabilizes Dicer (Paroo et al., 2009), and in the context of BDNF stimulation leads to increased levels of pre-miRNA processing.

While activity induced by BDNF may lead to increased processing of pre-miRNA, activity through glutamate receptors seems to accelerate mature miRNA turnover (Krol et al., 2010a). Using a variety of transcriptional inhibitors, Krol et al. (2010a) demonstrated that neuronal miRNAs have an unusually high turnover rate compared to other cell types. The turnover rate was further accelerated by addition of glutamate and decelerated by the application of to tetrodotoxin (TTX). These findings are strikingly similar to those made in *Aplysia* where application of serotonin results in the rapid degradation of miR-124 (Rajasethupathy et al., 2009). The mechanism for this rapid, activity-dependent miRNA turnover remains unclear. However, it is worth noting that at timepoints distal to neuronal activation, there are many more miRNAs that are downregulated than upregulated over a number of array-based studies (Eacker et al., 2011; Jimenez-Mateos et al., 2011; Griggs et al., 2013; Risbud and Porter, 2013). This activity-dependent miRNA turnover may allow neurons to rapidly reprogram RISC in a global manner in a way that promotes synaptic plasticity.

There is growing evidence that pre-miRNA processing can occur in dendrites. Biochemical purification of RISC components from synaptosomes showed detectable levels of pre-miRNA (Lugli et al., 2008). Recently, *in situ* hybridization methods that allow for specific detection of pre-miRNA have lent credence to the possibility that dendritically localized pre-miRNA may be an important phenomenon (Bicker et al., 2013). Using a clever biochemical approach, the authors of this study identified DHX36, a DExH-box RNA helicase as an interactor with the loop region of the dendritically localized pre-miR-134 transcript. Knockdown of DHX36 reduced dendritic transport of pre-miR-134 and enhanced the translation of reporters of miR-134 activity. The authors propose that DHX36 stabilizes pre-miR-134 for dendritic processing, perhaps in an activity-dependent manner, though this remains to be determined.

The *Drosophila *protein Armitage (Armi) and its mammalian homolog MOV10 are both implicated in the activity-dependent relief of miRNA repression (Ashraf et al., 2006; Banerjee et al., 2009). Armi and MOV10 are DExH-box RNA helicases that have been shown to be components of RISC (Tomari et al., 2004; Meister et al., 2005). In the case of *Drosophila*, Armi is degraded via the ubiquitin proteasome following activation of the nicotinic acetylcholine receptor. In mammals, MOV10 is also degraded in a proteosomal-dependent manner in response to NMDA receptor activation (Banerjee et al., 2009; Jarome et al., 2011). In neurons, degradation of MOV10/Armi relieves miRNA-mediated repression by an unknown mechanism, allowing for the translation of mRNAs involved in synaptic plasticity (Ashraf et al., 2006; Banerjee et al., 2009). Biochemical data concerning Armi’s function suggest that it is required for the loading of AGO with mature miRNAs following Dicer processing (Tomari et al., 2004). However, recent genome-wide studies have identified numerous promiscuous interactions between polyadenylated mRNAs and MOV10 (Castello et al., 2012; Sievers et al., 2012), suggesting additional potential interactions of MOV10 and miRNA-mediated silencing. More research is required to elucidate this potentially important interface between the relief of miRNA-mediated repression and activity-dependent protein synthesis.

## miRNAs FUNCTION IN DISEASES OF NEURONAL OVEREXCITATION

**FIGURE 3:**
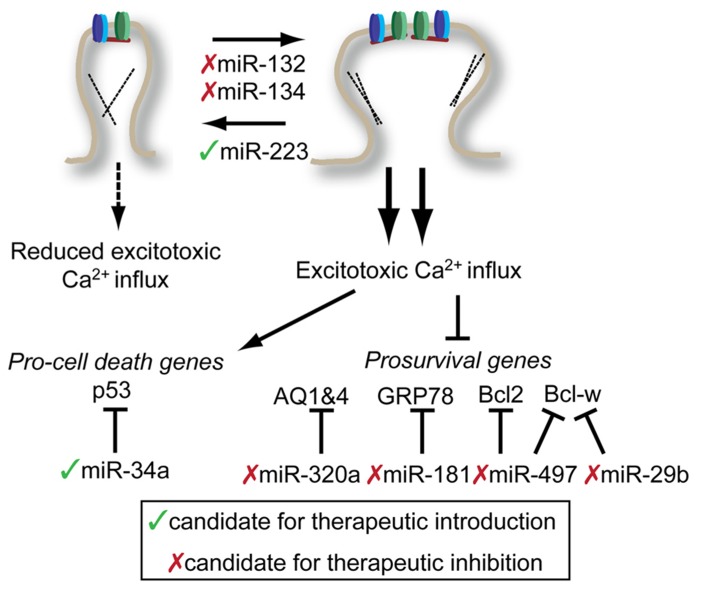
**Potential targets for miRNA-mediated therapies in the treatment of stroke and epilepsy.** Potential interventions target miRNAs to either reduce excitotoxic calcium influx (top) or reduce the impact of excessive calcium influx (bottom). Potential miRNA targets and be influenced either by introduction therapeutic inhibitors (red x) or mimics (green check). See text for details. Note: Conflicting results of miR-134 inhibition on spine morphology with embryonic neuronal studies described above. See text for details.

Excessive neuronal activity is associated with neuronal cell death in a number of contexts. During stroke, neuronal depolarization leads to excess glutamate release that cannot be compensated for by normal reuptake mechanisms. The resulting excess glutamate results in glutamate receptor hyperactivation and excitotoxic cell death via excess calcium influx. As miRNAs are potent regulators of the cellular stress response (Leung and Sharp, 2010; Mendell and Olson, 2012) and neuronal excitability (see above), they are a logical target for the investigation and enhancement of intrinsic neuroprotective pathways (**Figure 3**). A fair amount of effort has been made toward understanding the global miRNA response to stroke in rodent models. These studies generally rely on comparisons of RNA samples from stroke and sham brains, and subject them to some type of high-throughput array. Not surprisingly, the resulting miRNA expression profiles between different experimental stroke conditions, different laboratories, and different array platforms show almost no overlap. Despite this lack of overlap, there is valuable information that can be gained from a brief review of these studies.

One of the best studies that highlights the complexity of the miRNA response to CNS injury was performed on both blood and brain tissue from rats after ischemic stroke, intracerebral hemorrhage, or kainic acid-induced excitotoxicity (Liu et al., 2010). In this study, relatively few miRNAs showed consistent changes in expression in either brain tissue or blood. However, three miRNAs found in the blood (miR-155, miR-298, and miR-362-3p) change expression greater than twofold in response to some of the injuries. This suggests that expression of some miRNAs might be useful biomarkers to identify subtypes of CNS injuries.

In two related studies, the miRNA cortex of mice treated with middle cerebral artery occlusion (MCAO) model of stroke were profiled either by PCR 24 h post-stroke (Yin et al., 2010) or by array over an extended time course (Dharap et al., 2009). There was no significant overlap found between these two studies, perhaps because of methodological differences. Each study did identify some miRNAs that showed reproducible changes in their model. In one case, the authors found that miR-145 showed significant and enduring upregulation following stroke (Dharap et al., 2009). Inhibition of miR-145 with chemically modified antisense oligonucleotides (so-called antagomirs, or anti-miRs) lead to upregulation of superoxide dismutase 2, a predicted miR-145 target gene. Whether miR-145 inhibition had any therapeutic benefit was not determined. In the second study, the authors identified miR-497 as an upregulated miRNA in cortex following MCAO and in neuroblastoma cells subjected to oxygen–glucose deprivation (OGD), a cell culture model of stroke (Yin et al., 2010). Through a series of experiments, the authors show that Bcl-2 and Bcl-w, two anti-apoptotic molecules are targets of mIR-497. Infusion with antagomirs targeting miR-497 prior to MCAO resulted in reduced infarct volume and reduction in the severity of neurological deficits. Similar results were observed for mIR-29b, another miRNA which targets Bcl-w (Shi et al., 2012). The authors observed an increase in miR-29b following MCAO and showed that simple overexpression of miR-29b lead to spontaneous neuronal cell death in a Bcl-w-dependent manner.

Another approach toward finding potentially therapeutic miRNAs for treating stroke is to work backward from a known therapeutic target or pathway and identify miRNA interactors. GRP78 (also known as BIP) is a chaperone that is primarily localized to the ER and plays a key role in the ER-stress response. Ouyang et al. (2012) found that decreased GRP78 levels were accompanied by increased miR-181 in the core of MCAO-induced infarctions. After establishing that miR-181 could directly target GRP78, the author demonstrated that inhibition of mIR-181 through pretreatment with antisense oligo could significantly reduce infarct volume following MCAO. A similar approach identified miR-320a as a regulator of aquaporins 1 and 4 (AQ1 and AQ4) (Sepramaniam et al., 2010). Reduced expression of AQ1 and 4 in actrocytes is associated cerebral edema. By inhibiting miR-320a with antisense oligonucleotides, there was an increase in AQ1 and 4 expressions and a reduction in MCAO infarct volume.

Our lab recently investigated the role of miR-223 in neuroprotection motivated by the disproportionate number of nervous system targets predicted to be miR-223 targets (Harraz et al., 2012). Bioinformatic predictions identified that miR-223 should target glutamate receptor subunits NR2B and GluR2, making it a candidate for protection against excitotoxic insult. Targeted mutation of miR-223 leads increased levels of GluR2 and NR2B in the hippocampus, but not other post-synaptic proteins, and increased mEPSC decay time and amplitude. Consistent with these findings, miR-223 knockdown increased NMDAR-dependent calcium influx while overexpression decreased influx. Overexpression of miR-223 was protective across brain regions, protecting the striatum from NMDA-induced excitotoxicity and hippocampus from bilateral common carotid artery occlusion. Importantly miR-223 mutant mice were highly sensitized to both of these injuries, establishing a definitive role for miR-223 in the endogenous neuroprotective program.

Excitotoxicity also plays an important role in cell death associated with temporal lobe epilepsy (TLE), a chronic intractable form of epilepsy. TLE can be modeled in rodents by injection of pilocarpine, a muscarinic acetylcholine receptor agonist or injection of kainic acid, an agonist of the kainate-type glutamate receptor. Injection of either of these compounds results in establishment of *status epilepticus* (SE) in rodents. A number of studies have profiled miRNA from the hippocampus of mice following establishment of SE. Two studies from different research groups using kainic acid (Sano et al., 2012) and pilocarpine (Hu et al., 2012) model, respectively, found SE-associated upregulation of miR-34a in the hippocampus. MiR-34a expression is associated with p53-dependent pro-apoptotic program (Chang et al., 2007), making its upregulation by SE an attractive target for TLE therapy. Though both groups observed elevated caspase 3 cleavage associated with overexpression of miR-34a, only Hu et al. (2012) observed a protective effect by inhibiting miR-34a. Though methodological differences may explain some of the differences between these results, a robust and reproducible protective effect would be necessary to warrant further investigation of miR-34a in TLE therapeutics.

Two activity-regulated miRNAs, miR-132 and miR-134, are both upregulated in mouse models of TLE (Jimenez-Mateos et al., 2011, 2012). Interestingly, miR-132 induction in this TLE model is suppressed when mice are preconditioned with a low, peripherally administered dose of kainic acid (Jimenez-Mateos et al., 2011). This suggests that impairing miR-132 expression, by whatever means, may be neuroprotective. Indeed this was the case: inhibition of miR-132 by infusion of miR-132 antagomirs before kainic acid injection reduced cell death in the CA3 subfield of the hippocampus. Given the evidence from the synaptic plasticity field regarding miR-132’s ability to stimulate stable mushroom-type dendritic spines suggests that anti-miR-132 treatments may reduce cell death by reducing hippocampal neuron’s excitability, and therefore susceptibility to excitotoxicity. Similarly, miR-134 inhibition with antagomirs prior to SE induction provided neuroprotection. However, unlike miR-132 inhibition, a single injection of miR-134 antagomir provided long-lasting inhibition of recurrent spontaneous seizures (Jimenez-Mateos et al., 2012). Building on the work of Schratt et al. (2006), this study demonstrated that anti-miR-134 treatment reduced spine density *in vivo*, likely acting via Limk1. The long-lasting nature of the anti-miR-134 protection suggest that miR-134-based therapeutic may hold promise in treating intractable TLE.

## CONCLUSIONS AND FUTURE STUDIES

In the relative short time since their discovery, the miRNA field has made a dramatic impact on our understanding of nervous system function. Insights concerning the mechanisms of synaptic plasticity have been of particular interest, potentially coupling activity-dependent synaptic plasticity and protein synthesis. The local translation of proteins has long been considered a mechanism for regulating the connections at individual synapses and miRNA may be a key regulator at this point (Sutton and Schuman, 2006). However attractive this hypothesis is, there have been no clear demonstrations that miRNA can regulate local, activity-dependent protein synthesis. Furthermore, while there is some evidence that miRNA can sequester mRNAs from translating ribosomes, it is not clear that sequestered mRNA can undergo reactivation. In some studies, investigators observe a decrease in miRNA-targeted mRNA levels consistent with the deadenylation and decay model while other see only a change in protein levels, more consistent with a sequestration model. While a difficult problem to interrogate, understanding local control of protein synthesis by miRNA should be a central focus of future investigations.

As discussed above, individual miRNAs can have potentially hundreds of targets owing to the small amount of complementarity required to establish miRNA–mRNA interactions. In virtually all studies, there is a focus on a one miRNA–one target interaction. This is largely for practical purposes – requirements for publication dictate a laser-like focus on a making a bulletproof set of observations. While editors and reviewers require this type of focus, miRNAs do not have such single-minded focus (Baek et al., 2008; Selbach et al., 2008). Having a more comprehensive view of how individual miRNAs regulate the proteome will be necessary, especially if they are to be used in therapeutics.

Virtually all the studies described in this review rely on antisense oligonucleotides or antagomirs to conduct loss-of-function experiments. While these reagents are widely used and proven effective under many circumstances, virtually nothing is known about their distribution and perdurance in the CNS following intracranial injection, making interpretation of how they work problematic. A reasonable complement to antagomir application would the use of traditional targeted mutations, which are becoming increasingly more available to the general scientific research world (Park et al., 2012).

Finally, the most successful candidate miRNAs for translation to the clinic have arisen from rigorous work in the basic sciences. Both miR-132 and miR-134 have a long track record of careful investigation in the basic synaptic plasticity field and have now been shown to have potential in the treatment of epilepsy. This cross-pollination of basic and translational science seems to be the most fruitful way forward for the future of miRNA-based therapeutics.

## Conflict of Interest Statement

The authors declare that the research was conducted in the absence of any commercial or financial relationships that could be construed as a potential conflict of interest.
